# Doege–Potter syndrome due to endothoracic solitary hypoglycemic fibrous tumor

**DOI:** 10.1002/ccr3.5611

**Published:** 2022-04-04

**Authors:** Madaleine Lopez‐Hinostroza, Jeel Moya‐Salazar, Juan Dávila, Angélica Y. Absencio, Hans Contreras‐Pulache

**Affiliations:** ^1^ Department of Respiratory Disease Hospital Nacional Guillermo Almenara Irigoyen Lima Peru; ^2^ Hospital Nacional Docente Madre Niño San Bartolomé Lima Peru; ^3^ Cancerology and Pathology Unit NESH Hubbs Lima Perú; ^4^ Digital Transformation Center Norbert Wiener University Lima Perú; ^5^ Department of Pathology Hospital Nacional Guillermo Almenara Lima Perú; ^6^ 33213 South America Center for Education and Research in Public Health Universidad Norbert Wiener Lima Peru

**Keywords:** Doege–Potter syndrome, hypoglycemia, insulin‐like growth factor II, non‐islet cell tumor, Peru, solitary fibrous tumor

## Abstract

Doege–Potter syndrome leads to severe and sustained symptomatic hypoglycemia and is associated with the solitary fibrous tumor. It is a rare cause, and its diagnosis requires a clinical suspicion and other markers such as insulin‐like growth factor II. Here, we describe a case of a patient with intrathoracic tumor and hypoglycemia.

## Graphical abstract

Doege–Potter syndrome is a rare cause of symptomatic, severe, and sustained hypoglycemia. This syndrome is associated with a solitary fibrous tumor, and its diagnosis is based on an acute set of clinical characteristics and biological markers.

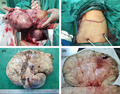

## 1 INTRODUCTION

In 1930, Karl Doege and Roy Potter described the presence of a non‐beta‐pancreatic cell tumor associated with hypoglycemia in a patient with a fibrous tumor in the mediastinum. Since then, the eponymous phrase of these authors has been used to refer to the presence of an intrathoracic tumor associated with symptomatic, severe, and sustained hypoglycemia. The pathophysiological mechanisms that explain the hypoglycemic syndrome are glucose consumption by the tumor and excess secretion of insulin‐like growth factor II (IGF‐II), a protein that inhibits the release of glucose by the liver, and substances that inhibit its action or the secretion of counterinsular hormones, which lead to the failure of one of the mechanisms to prevent hypoglycemia.^1^

The solitary fibrous tumor (SFT) is a slow‐growing, large tumor of mesenchymal origin that can cause Doege–Potter syndrome in about 2%–4% of cases.^2^ SFT is a slow‐growing, large tumor of mesenchymal origin that can cause Doege–Potter syndrome in about 2%–4% of cases. SFT is difficult to distinguish from other tumors. On chest tomography, it generally appears as a round and well‐defined homogeneous mass, large tumors can reach more than 20 cm in diameter, and 90% of them have non‐malignant features.^3^

For its diagnosis, immunohistochemical markers such as cluster differentiation (CD) CD34 are used, which is strongly expressed by this tumor, to differentiate it from other cancerous tumors. Complete surgical resection to negative margins, even for tumors classified as high risk, given the low global metastatic potential and the lack of efficacious adjuvant therapy, is the mainstay of localized SFT therapy.^3^, ^4^

Here, we describe for the first time an interesting case of a Peruvian patient who accidentally discovered an intrathoracic tumor of mesenchymal origin, accompanied by severe and persistent hypoglycemia.

## 2 CASE REPORT

A 74‐year‐old male patient with a history of hypertension 15 years ago, type 2 diabetes mellitus 20 years ago, and metformin 850 mg PO q12hr treatment presents with mMRC2 grade dyspnea, chest pain that has not radiated for 2 weeks before admission. Due to sudden loss of consciousness, he is taken to emergency, being immediately referred to the Shock Trauma Unit (Glasgow 10/15), to control vital functions: blood pressure (BP): 170/90 mmHg; heart rate (HR): 90 bpm; respiration rate (RR): 26; blood oxygen saturation (SpO2): 95%; fraction of inspired oxygen (FiO2): 21%; and on preferential clinical examination: vesicular murmur found abolished in the left hemithorax and preserved in the right hemithorax, and no added sounds are heard.

**FIGURE 1:**
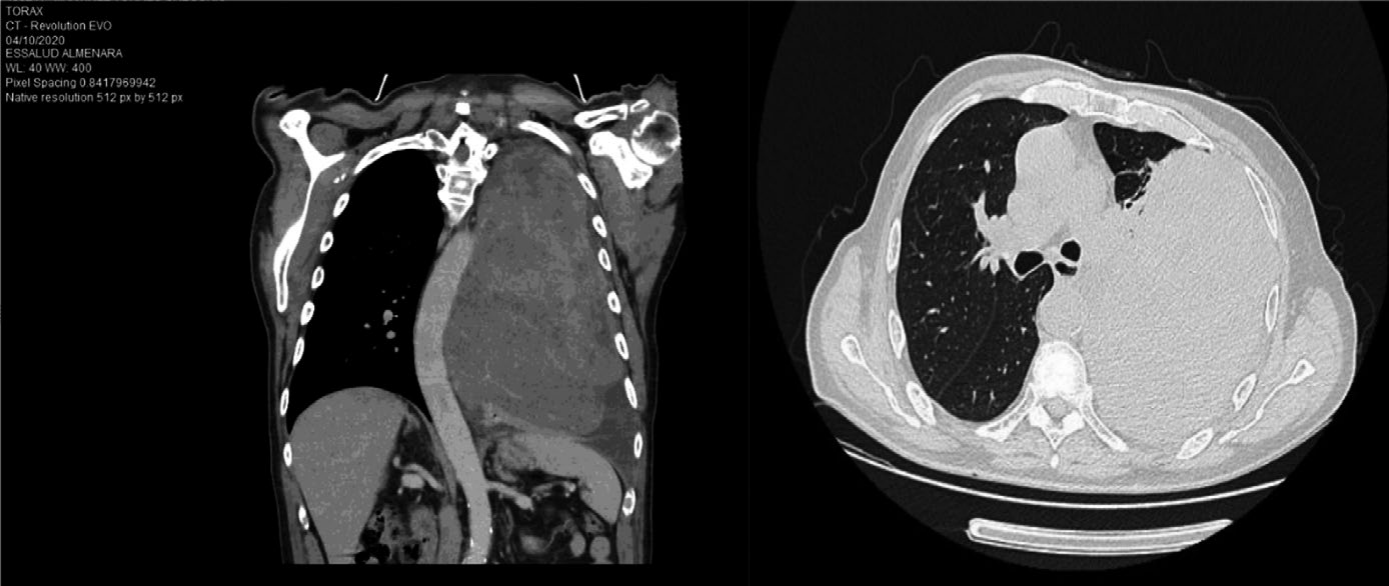
Coronal section chest CT scan shows a mass with a neo‐proliferative appearance of the left lung measuring 24.9 cm × 15.8 cm × 18.9 cm, associated with ipsilateral laminar pleural effusion and mass effect on the contralateral mediastinal structures

We took capillary blood glucose (38 mg/dl), classifying it as acute encephalopathy without neurological targeting and symptomatic hypoglycemia. Glucose infusion serum treatment was administered, recovering consciousness without any neurological sequelae. Computerized axial tomography of the chest without contrast was performed, showing a mass and left pleural effusion (Figure 1). The patient in his serial capillary glycemia controls presented episodes of symptomatic, sustained, and persistent hypoglycemia, for which treatment with glucose serum was maintained. In hematic biometry controls, we found a glycosylated hemoglobin (HbA1c): 5.7% [normal range (NR) 4%–5.6%], basal cortisol 8am of 5.9 mcg/dl (NR: 5–23 mcg/dl), insulin‐like growth factor I (IGF I): 74 ng/ml (NR: 50–160 ng/ml), peptide C: 0.01 ng/dl (NR: 0.5–2.0 ng/dl), and basal insulin of 0.04 mIU/L (NR: <25 mIU/L).

## 3 INVESTIGATION

**FIGURE 2:**
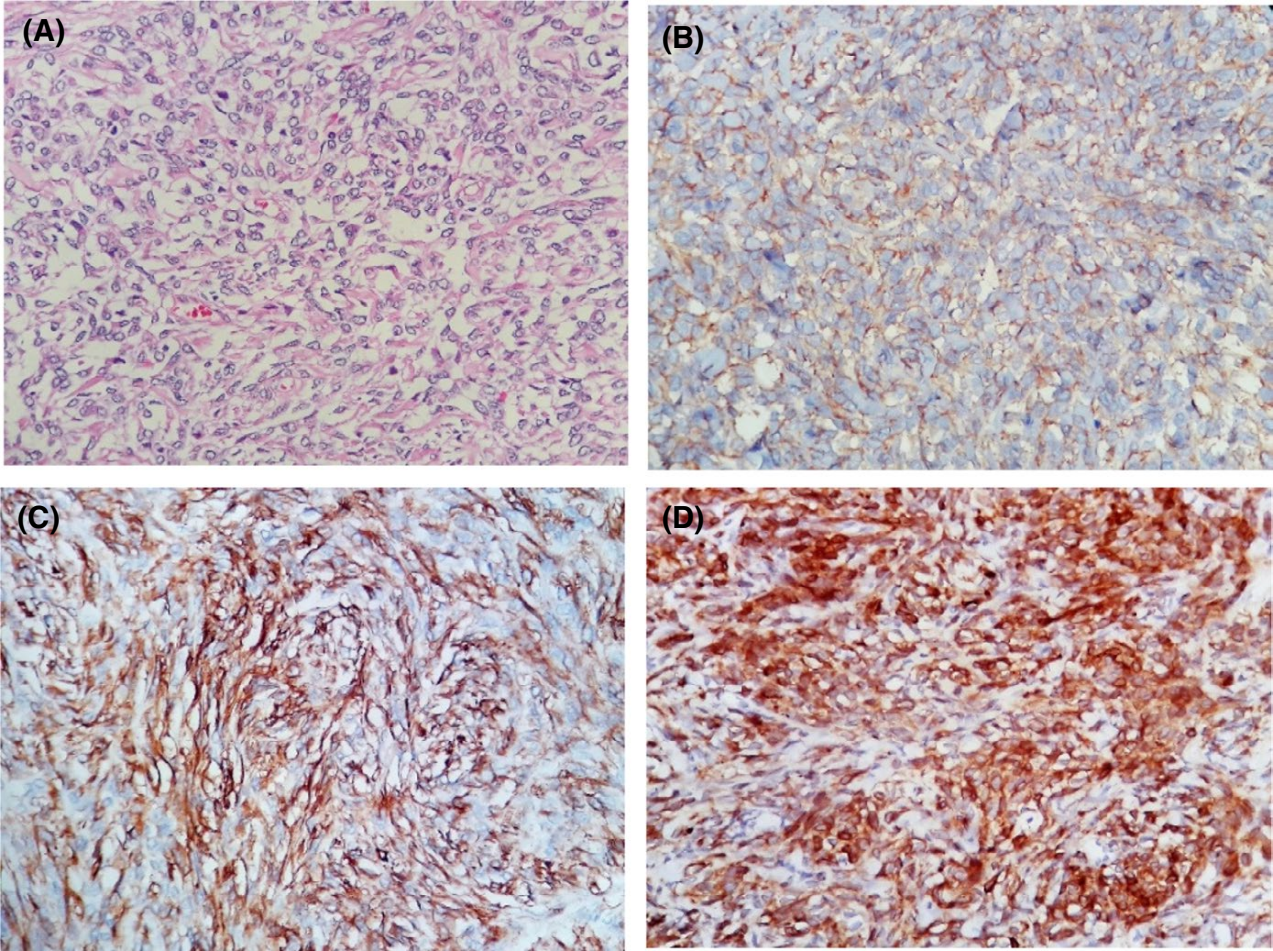
Solitary fibrous tumor, microscopy. Microscopy revealed a storiform pattern arranged in hypocellular and hypercellular areas, necrosis of 20% of the tumor, and 6 mitoses in 10 high‐power fields. With the immunohistochemical markers, it was possible to observe positivity for CD99, CD34, BCL‐2, with actin and S100 being negative. With these findings, the final diagnosis was a malignant solitary fibrous tumor. (A) Storiform spindle cell proliferation, (B) CD99 positive, (C) CD34 positive, (D) Bcl‐2 positive (all in 100×)

A bronchofibroscopy was performed, finding direct signs of malignancy and stenosis of the left bronchial branch. For this reason, transbronchial biopsy samples of the left lung mass and bronchial lavage were taken, obtaining an SFT. The tumor immunohistochemistry results were B‐cell lymphoma 2 (BCL‐2) positive, focal CD34 +, focal cytoplasmic CD99+, epithelial membrane antigen (EMA) and Panker negative, actin negative, S100 protein‐negative, Papanicolaou and Block cell with a positive result for malignant tumor cells (Figure 2). In addition, bone scintigraphy was performed with a negative test result for metastasis.

The patient continues to present with symptomatic and recurrent hypoglycemia, despite considering endogenous hyperinsulinism in the differential diagnosis. Since the IGF II value was found to be 721 U/I, we considered non‐island cell hypoglycemia and paraneoplastic syndromes of IGF‐II producing tumors as a definite diagnosis.

**FIGURE 3:**
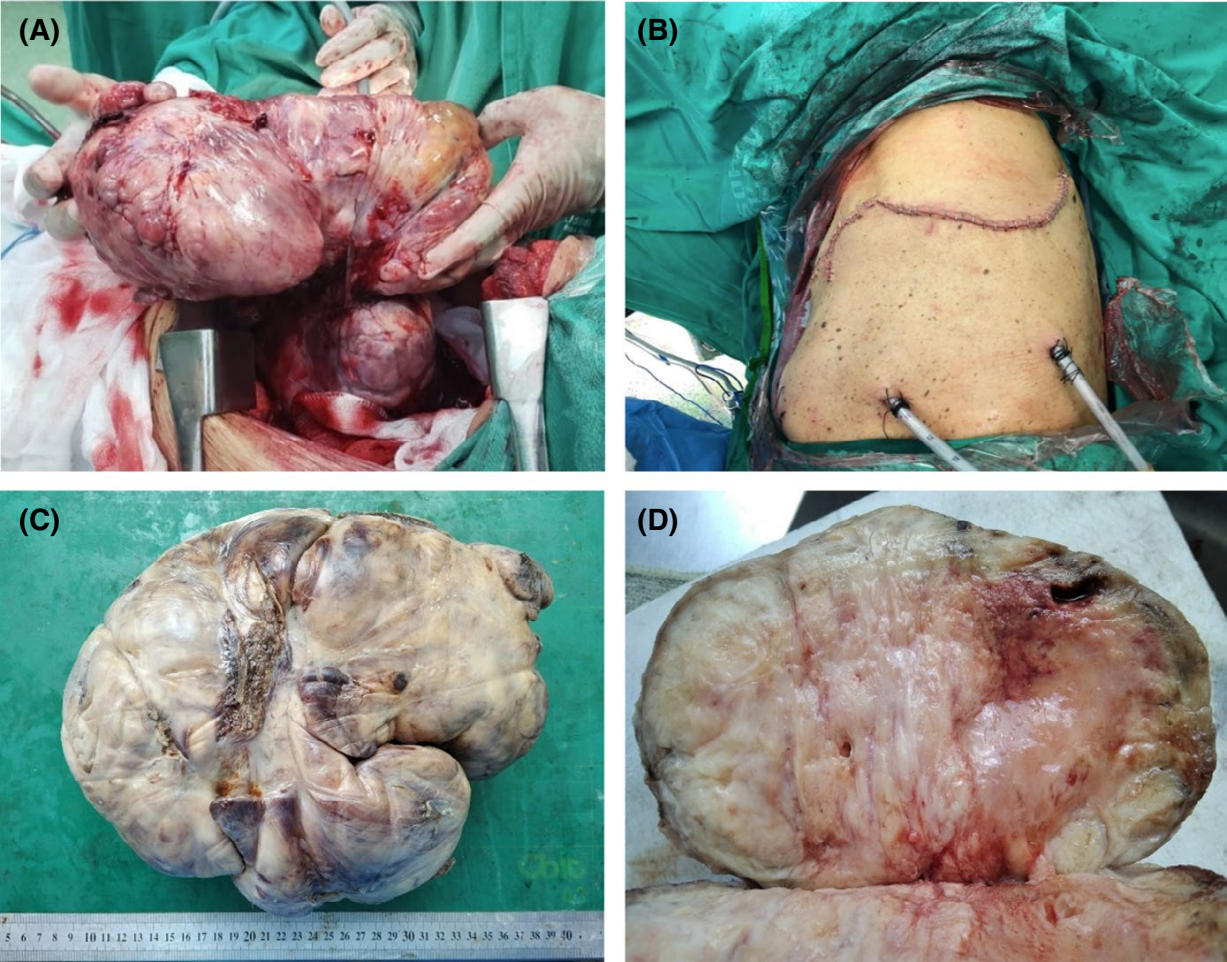
Surgery processes. (A) complete removal of solitary fibrous tumor (B) placement of 02 anterior and posterior TDT in the left hemithorax. Solitary fibrous tumor, macroscopy. (C) Solid tumor (25 × 20 × 15 cm) lobulated weighing 3.1 kg, areas of necrosis and hemorrhage were identified in the laminations. (D) Solid whitish‐brown cut surface, with areas of necrosis and hemorrhage

A pulmonary ventilation/perfusion scintigraphy was performed, indicating the absence of left lung perfusion and preservation of the right lung. A multidisciplinary medical meeting was held deciding to excise the lung tumor by the chest surgery team. Surgical intervention was performed 31 days after admission, and the operating time was 11 hours. Complete removal of the endothoracic tumor (weight: 3.1 kg) was successfully achieved, which was successful and sent for a pathological study, and two left thoracic drainage tubes were placed: anterior and posterior (Figure 3).

**FIGURE 4:**
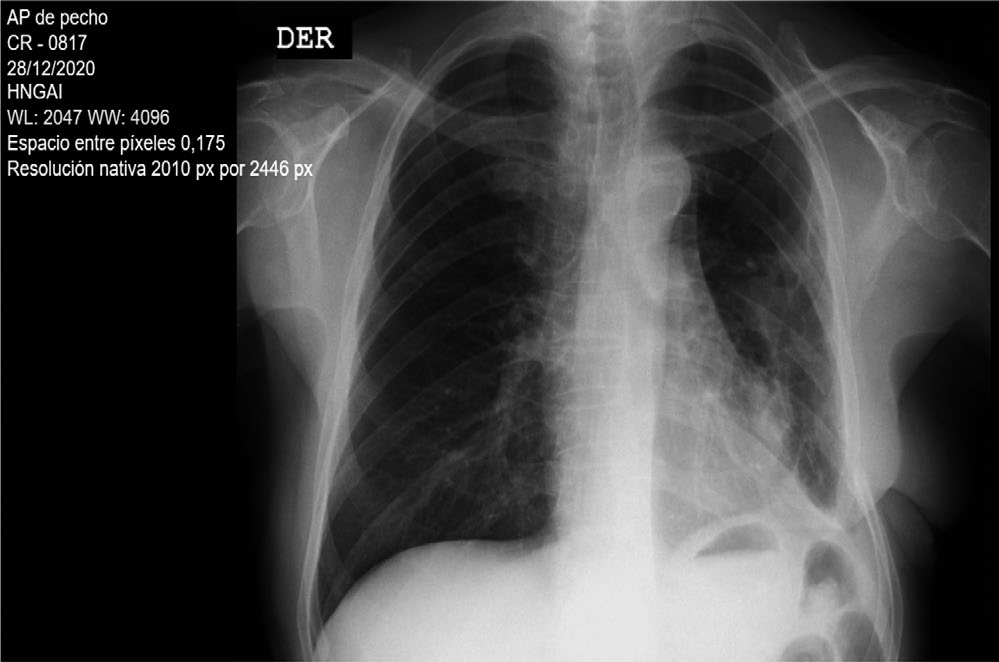
Control chest X‐ray 3 weeks after discharge, showing lung expansion

On the same day, a few hours later, the patient went to cardiorespiratory arrest with asystole rhythm due to type III and IV acute respiratory failure and presented hemorrhagic shock. Cardiopulmonary resuscitation was performed for 2 minutes with the administration of vasoconstrictor drugs and a transfusion of 4 concentrated blood cells. The patient responded to extubating 2 days after the operation, and there were no sequelae to the cardiorespiratory arrest. Five days later, both chest drainage tubes were removed. On the sixth day after surgery, the patient was discharged without re‐presenting hypoglycemic episodes. On evaluation by outpatient consultation 3 weeks after discharge, the chest radiograph shows pulmonary re‐expansion in the left hemithorax (Figure 4).

## 4 DISCUSSION

This report is one of the few in Peru^5^ and agrees with the clinical presentation reported by Han et al.,^4^ who state that the most common cause of Doege–Potter syndrome is a benign SFT involving the right hemithorax associated with pleural damage. Han et al., give an account 71 cases of Doege–Potter syndrome, 43 of which were malignant (60.6%), including SFT of the pelvis, bladder, and retroperitoneum associated with high‐frequency hypoglycemia.^4^

The SFT associated with Doege–Potter syndrome reported in this 74‐year‐old patient has demonstrated positivity for BCL‐2 +, focal CD34 +, and focal CD99 +. Mohammed et al.^6^ reported a considerable number of positive lesions for CD34 + and CD99 +, and other case reports have shown immunoreactivity for these cellular markers.^5^, ^7^ However, the authors emphasize that these immunohistochemical markers do not have high specificity and may be present in many soft tissue neoplasms.

In this study, we reported negative for actin, S100, and EMA, which is different from previous reports that showed positive for smooth muscle actin, EMA, S100, and keratin. Given this unusual performance of these markers, The Signal transducer and activator of transcription 6 (STAT6) has turned out to be more specific, being positive in >95% of the cases, recently reported as part of the pool of tests to address these cases.^8^

This clinical case reported Doege–Potter syndrome secondary to the SFT tumor. However, the main paraneoplastic symptom of SFT is osteoarthropathy, hypoglycemia is less than 5%, which is a rare complication. Hypoglycemia is usually secondary to the tumor, overproducing IGF II precursor that leads to tumor growth, malignant transformation, and carcinogenesis.^9^ Our patient had an IGF II concentration of 721 U/I that exceeds the threshold of 10 U/I, indicating an excess production of IGF II characteristic of non‐islet cell tumors.^4^, ^9^, ^10^

Insulin is a 51‐amino acid protein with hormone functions and has mediators such as peptide C in its production process. This hormone is responsible for glycemic control, and in Doege–Potter syndrome, it is common for insulin levels to be decreased. As in previous studies,^6^, ^7^ our patient had low levels of basal insulin (0.04 U/I) and C‐peptide (0.01 ng/dl).

Among the euglycemic disorders, patients with diabetes mellitus may develop hypoglycemia.^11^ Hypoglycemia is usually iatrogenic, linked to poor therapeutic management or rarely prolonged‐time without glycemic intake.^12^ Certain tumors can lead to hypoglycemia. The most common hypoglycemia‐inducing tumors are insulinomas, neuroendocrine and mesenchymal tumors, and ovarian carcinomas. Some tumors produce autoantibodies against insulin or the insulin receptor that cause hypoglycemia in the paraneoplastic setting.^13^

The most frequent age of SFT is older adults. Several studies have reported SFT in patients aged 67 and 83 years,^6^, ^9^, ^14^ although there are case series in patients aged 48 years,^15^ 52 years,^8^ 55 years,^7^ and 58 years.^3^ In this study, an elderly patient (74‐year‐old) with Doege–Potter syndrome secondary to benign SFT was reported for the first time in Peru, which is different from previous reports.^7^

The definitive underlying treatment for Doege–Potter syndrome secondary to benign SFT is palliative debulking, a complete resection of the tumor mass, chemotherapy, cryoablation, radiofrequency ablation, or chemoembolization. Previous studies have described complete resection as an effective treatment for this disease, as seen in some cases, including the one we reported in this article.^4^, ^6^, ^9^ In addition, other surgical techniques with a favorable prognosis have been reported as successful.^3^, ^16^

With the discovery of the NAB2‐STAT6 gene fusion as the characteristic molecular driver of SFT, embolization therapy, and radiation be effective. Based on this, individualized therapies aimed at inhibiting the descending pathways of NAB2‐STAT6 could be considered as the treatment of this rare group of neoplasms.^6^

Finally, there is a growing interest in SFT and Doege–Potter syndrome. In Latin America, several cases have been reported in the Argentinian,^17^, ^18^ Peruvian,^7^ Chilean,^19^ Brazilian,^8^ Colombian,^5^, ^14^, ^20^, ^21^ and Bolivian^22^ population, which demonstrates that even being a rare cause of the paraneoplastic syndrome is of great interest in clinical practice.

## 5 CONCLUSION

Doege–Potter syndrome associated with a solitary fibrous tumor is a rare cause of paraneoplastic complication. The answer to the diagnosis is IGF II quantification, the glycemic control profile, and immunohistochemical markers. Surgery has been effective with a favorable prognosis at follow‐up.

## CONFLICT OF INTEREST

The authors declare that there is no conflict of interest regarding the publication of this article.

## AUTHOR CONTRIBUTIONS

All authors contributed equally to this work.

## ETHICAL APPROVAL

The patients described were fully informed on the method and the purpose of the case report. Written consent to participate and for publication was obtained by the patients and is available upon request.

## CONSENT

All authors have confirmed during submission that patients' consents have been signed and collected in accordance with the journal's patient consent policy. Written informed consent was obtained from the patient to publish this report in accordance with the journal's patient consent policy.

## Data Availability

The data that support the findings of this study are available from the corresponding author upon reasonable request.
